# Lifetime self-reported arthritis is associated with elevated levels of mental health burden: A multi-national cross sectional study across 46 low- and middle-income countries

**DOI:** 10.1038/s41598-017-07688-6

**Published:** 2017-08-02

**Authors:** Brendon Stubbs, Nicola Veronese, Davy Vancampfort, Trevor Thompson, Cristiano Kohler, Patricia Schofield, Marco Solmi, James Mugisha, Kai G. Kahl, Toby Pillinger, Andre F. Carvalho, Ai Koyanagi

**Affiliations:** 1 South London and Maudsley NHS Foundation Trust, Denmark Hill London, SE5 8AZ United Kingdom; 2 Institute of Psychiatry, Psychology and Neuroscience, King’s College London, De Crespigny Park London, Box SE5 8AF United Kingdom; 3 National Research Council, Neuroscience Institute, Aging Branch, Padova, Italy; 4 Institute of clinical Research and Education in Medicine (IREM), Padova, Italy; 5 KU Leuven Department of Rehabilitation Sciences, Leuven, Belgium; 6 KU Leuven, University Psychiatric Center KU Leuven, Leuven-Kortenberg, Belgium; 7 Faculty of Education and Health, University of Greenwich, London, United Kingdom; 8 Department of Clinical Medicine and Translational Psychiatry Research Group, Faculty of Medicine, Federal University of Ceará, Fortaleza, CE Brazil; 9 Faculty of Health, Social Care and Education, Anglia Ruskin University, Chelmsford, United Kingdom; 10 Department of Neurosciences, University of Padova, Padova, Italy; 11 Kyambogo University, Kampala, Uganda; 12 Butabika National Referral and Mental Health Hospital, Kampala, Uganda; 13 Department of Psychiatry, Social Psychiatry and Psychotherapy, Hannover Medical School, Hannover Medical School, Hannover, Germany; 14 Department of Clinical Medicine and Translational Psychiatry Research Group, Faculty of Medicine, Federal University of Ceará, Fortaleza, Brazil; 15 Research and Development Unit, Parc Sanitari Sant Joan de Déu, Universitat de Barcelona, Fundació Sant Joan de Déu, Dr. Antoni Pujadas, 42, Sant Boi de Llobregat, Barcelona, 08830 Spain; 16 Instituto de Salud Carlos III, Centro de InvestigaciónBiomédicaenRed de Salud Mental, CIBERSAM, Monforte de Lemos 3-5 Pabellón 11, Madrid, 28029 Spain

## Abstract

Population-based studies investigating the relationship of arthritis with mental health outcomes are lacking, particularly among low- and middle-income countries (LMICs). We investigated the relationship between arthritis and mental health (depression spectrum, psychosis spectrum, anxiety, sleep disturbances and stress) across community-dwelling adults aged ≥18 years across 46 countries from the World Health Survey. Symptoms of psychosis and depression were established using questions from the Mental Health Composite International Diagnostic Interview. Severity of anxiety, sleep problems, and stress sensitivity over the preceding 30 days were self-reported. Self-report lifetime history of arthritis was collected, including presence or absence of symptoms suggestive of arthritis: pain, stiffness or swelling of joints over the preceding 12-months. Multivariable logistic regression analyses were undertaken. Overall, 245,706 individuals were included. Having arthritis increased the odds of subclinical psychosis (OR = 1.85; 95%CI = 1.72–1.99) and psychosis (OR = 2.48; 95%CI = 2.05–3.01). People with arthritis were at increased odds of subsyndromal depression (OR = 1.92; 95%CI = 1.64–2.26), a brief depressive episode (OR = 2.14; 95%CI = 1.88–2.43) or depressive episode (OR = 2.43; 95%CI = 2.21–2.67). Arthritis was also associated with increased odds for anxiety (OR = 1.75; 95%CI = 1.63–1.88), sleep problems (OR = 2.23; 95%CI = 2.05–2.43) and perceived stress (OR = 1.43; 95%CI = 1.33–1.53). Results were similar for middle-income and low-income countries. Integrated interventions addressing arthritis and mental health comorbidities are warranted to tackle this considerable burden.

## Introduction

Recent global burden of disease surveys have demonstrated that chronic musculoskeletal and joint conditions are leading causes of disability, particularly in Western societies^1, 2^. One of the main clusters of chronic musculoskeletal and joint disorders is arthritis, a broad term encompassing osteoarthritis (OA) and inflammatory arthritic conditions such as rheumatoid arthritis (RA). The hallmark features of arthritis in both OA and RA are pain and discomfort. Unsurprisingly, an increasing body of evidence has demonstrated that OA^3^ and RA^4^ are associated with high levels of disability and lower quality of life^5^.

Recently, there is increasing interest in the mental health burden of arthritis. Specifically, Matcham *et al*.^6^ in a meta-analysis of 72 studies established that 38% of people with RA met the criteria for depression according to the Patient Health Questionnaire^7^ whilst 16.8% had major depressive disorder, figures considerably higher than the general population. The presence of anxiety and depression in RA is important since it is known to predict treatment response in patients with RA^8^. Stubbs *et al*.^9^ recently conducted a meta-analysis and established that one fifth of people with OA had anxiety or depression. A recent longitudinal study in North America demonstrated that multisite OA is associated with an increased incidence of depression^10^. A number of single country studies have also demonstrated that RA^11^ and OA^12^ are associated with sleep disturbance. Sleep disturbance and perceived stress have also been demonstrated to be associated with worse outcomes in people with RA^13^. Thus, clearly, mental health symptoms are common and negatively impact the quality of life and treatment outcomes for people with arthritis.

Whilst progress has been made in understanding the mental health burden associated with arthritis, some pertinent limitations and gaps within the literature exist. First, to date, most studies considering arthritis and mental health have been based on clinical samples, and there is a lack of community-based studies. Moreover, there is a paucity of large representative multinational studies, particularly among low- and middle-income countries (LMICs). The majority of the world’s population resides in LMICs. A recent meta-analysis demonstrated that 3.16 million males and 14.87 million females were affected by RA in 2010 in LMICs with a rapid increase expected^14^. Resources to deal with the physical aspect of arthritis or mental health generally are not well established nor a priority in LMICs. Therefore, this comorbidity may be particularly challenging in this setting, and understanding the mental health burden of arthritis is important for planning service development. Moreover, people in LMICs are more likely to undertake labor-demanding jobs in the informal sector with no job security or compensation for lost income. Therefore, maintaining good mental and physical health is crucial for their livelihoods and general welfare. Furthermore, it is possible, for example, that pain associated with arthritis could increase the mental health burden and stress in an environment where reliance on income from labor-demanding jobs is widespread. Therefore, there is a need to elucidate the potential mental health burden among people with arthritis in LMICs. Second, the majority of studies considering mental health comorbidity and arthritis have focused on common mental disorders such as depression or anxiety and very few studies have considered psychosis for instance. However, there is an intriguing negative relationship that has been reported between schizophrenia and RA in Western counties^15^ and a paucity of data is available on arthritis and people with psychotic symptoms who do not meet the criteria for a diagnosis. In addition, there is increasing recognition that depression sits on a continuum with various subtypes, yet there is a lack of studies considering the relationship between arthritis and the depression continuum. Clearly, understanding a wider range of mental health comorbidities (including depression subtypes, psychosis, sleep disturbance, anxiety, and perceived stress) in arthritis important.

Given the aforementioned, the aim of the current study was to explore the relationship between arthritis and mental health (depression subtypes, psychosis, anxiety, sleep problems, and stress sensitivity). We hypothesized that people with arthritis in both low-income and middle-income countries would have worse mental health outcomes than those without arthritis.

## Data and Methods

### Procedures

The data for the current study was captured from the World Health Survey (WHS). The WHS was a cross-sectional study undertaken in 2002–2004 in 70 countries worldwide. Data were collected using single-stage random sampling and stratified multi-stage random cluster sampling across 10 and 60 countries respectively. Full details of the WHS are available elsewhere (http://www.who.int/healthinfo/survey/en/). In brief, persons aged ≥18 years with a valid home address were eligible to participate. Each member of the household had equal probability of being selected by utilizing Kish tables. A standardized questionnaire, translated accordingly was used across all countries. Linguists ensured that the translation was conducted to a high standard. The individual response rate (i.e. ratio of completed interviews among selected respondents after excluding ineligible respondents from the denominator) ranged from 63% (Israel) to 99% (Philippines)^16^. Ethical approval to conduct this study was obtained from the ethical boards at each study site (see Appendix 1 for details of approving board at each study site) and in accordance with each sites regulations. Sampling weights were generated to adjust for non-response and the population distribution reported by the United Nations Statistical Division. Informed consent was obtained from all participants.

Of the 70 countries, 69 had data which were publically available. Of these, 10 countries (Austria, Belgium, Denmark, Germany, Greece, Guatemala, Italy, Netherlands, Slovenia, and UK) were excluded due to lack of data on sampling information. Furthermore, 10 high-income countries (Finland, France, Ireland, Israel, Luxembourg, Norway, Portugal, Spain, Sweden, United Arab Emirates) were excluded in order to focus on LMICs. Of the remaining LMICs, Slovakia, Congo, and Swaziland were excluded as >25% of the data on arthritis was missing. Thus, the final sample consisted of 46 countries which corresponded to 20 low-income and 26 middle-income countries according to the World Bank classification at the time of the survey (2003).

### Primary variables

#### Arthritis (exposure variable)

Individuals with a self-reported lifetime diagnosis of arthritis and/or typical symptoms of arthritis were considered to have arthritis. The specific question used to assess a lifetime diagnosis was “Have you ever been diagnosed with arthritis (a disease of the joints)”?

We also used a symptom-based approach to minimize reporting bias especially in areas where access to medical facilities is limited. The symptom-based algorithm was based on questions on typical clinical symptoms used in previous publications using the same questionnaire^17, 18^. Specifically, those who replied affirmatively to both of the following questions were considered to have arthritis: During the last 12 months, have you experienced: (a) pain, aching, stiffness or swelling in or around the joint (like arms, hands, legs or feet) which were not related to an injury and lasted for more than a month?; (b) stiffness in the joint in the morning after getting up from bed, or after a long rest of the joint without movement?

### Mental health conditions (outcome variables)

#### Depression type

The severity of depressive symptoms was established based on the individual questions of the World Mental Health Survey version of the Mental Health Composite International Diagnostic Interview (CIDI), which assessed the duration and persistence of depressive symptoms in the past 12 months^19^. Following the algorithms used in a previous WHS publication^20^, four mutually exclusive groups were established based on the ICD-10 Diagnostic Criteria for Research (ICD-10-DCR) where criterion B referred to symptoms of depressed mood, loss of interest, and fatigability. The algorithms used to define the four groups were the following: (a) Depressive episode group: At least two criterion B symptoms together with a total of at least four depressive symptoms lasting two weeks most of the day or all of the day. (b) Brief depressive episode group: Same criteria as depressive episode above but duration did not meet the two-week duration criterion. (c) Subsyndromal depression: At least one criterion B symptom together with the total number of symptoms being three or less. The criteria of duration of at least two weeks and presence of symptoms during most of the day had to be met. (d) No depressive disorder group: None of the above.

### Psychosis

Participants were asked whether they had ever been diagnosed as having schizophrenia or psychosis. All participants, regardless of a psychosis diagnosis, were asked questions on positive psychotic symptoms which came from the WHO Composite International Diagnostic Interview(CIDI) 3.0^19^. This psychosis module has been reported to be highly consistent with clinician ratings with a kappa agreement coefficient of 0.82 for DSM-IV diagnosis of schizophrenia with even higher concordance observed for hallucinations and delusions^21^. Furthermore, psychotic experiences determined with the CIDI have been reported to be a good screening tool to identify those at high risk of developing psychosis^22^. The hallucinations question excluded conditions associated with sleep-related states or substance use. Specifically, respondents were asked the following questions with answer options ‘yes’ or ‘no’: During the last 12 months, have you experienced (i) ‘A feeling something strange and unexplainable was going on that other people would find hard to believe’? (delusional mood); (ii) ‘A feeling that people were too interested in you or there was a plot to harm you’? (delusions of reference and persecution); (iii) ‘A feeling that your thoughts were being directly interfered or controlled by another person, or your mind was being taken over by strange forces’? (delusions of control); (iv) ‘An experience of seeing visions or hearing voices that others could not see or hear when you were not half asleep, dreaming or under the influence of alcohol or drugs’? (hallucinations).

Individuals who endorsed at least one of the four above-mentioned psychotic symptoms were considered to have psychotic symptoms. Based on information on psychosis diagnosis and psychotic symptoms, a three-category psychosis variable was constructed: (i) no psychosis diagnosis and no psychotic symptoms (control); (ii) at least one psychotic symptom but no psychosis diagnosis (subclinical psychosis); and (iii) psychosis diagnosis^23, 24^.

### Sleep problems

Sleep problems were assessed by the question “Overall in the last 30 days, how much of a problem did you have with sleeping, such as falling asleep, waking up frequently during the night or waking up too early in the morning”? with answer options none, mild, moderate, severe, and extreme. Those who answered severe and extreme were considered to have sleep problems. This definition has been used in previous publications using the same survey question on sleep problems^18, 25, 26^.

### Anxiety

Anxiety was assessed by the question “Overall in the past 30 days, how much of a problem did you have with worry or anxiety”? Respondents could answer: none, mild, moderate, severe, or extreme. In the current study those who answered severe and extreme were categorized as having anxiety^26, 27^.

### Perceived stress

Perceived stress in the last month was assessed by two questions: “How often have you felt that you were unable to control the important things in your life”?; and “How often have you found that you could not cope with all the things that you had to do?” The answer options to these questions were: never (score = 1), almost never (score = 2), sometimes (score = 3), fairly often (score = 4), very often (score = 5). The scores of the two questions were added to create a scale ranging from 2 to 10^28^. The highest quintile (cut-off ≥7) was used to define a high level of perceived stress.

### Other variables

Variables on sex, age, highest education achieved (no formal education, primary education, secondary or high school completed, or tertiary education completed), wealth, setting (rural or urban), smoking, alcohol consumption, angina, asthma, diabetes, and Body Mass Index (BMI) were considered as potential correlates of arthritis based on past literature^4, 29^. Principal component analysis based on 15–20 assets was performed to establish country-wise wealth quintiles. The question on smoking was ‘Do you currently smoke any tobacco products such as cigarettes, cigars, or pipes’? with the answer options being ‘daily’, ‘yes, but not daily’, or ‘no, not at all’. This variable was dichotomized into those who smoked regardless of frequency (i.e. daily or not daily) (current smokers) and those who do not smoke. Alcohol consumption was assessed by first asking the question ‘Have you ever consumed a drink that contains alcohol (such as beer, wine, etc.)’? Respondents who replied ‘no’ were considered lifetime abstainers. If the respondent replied affirmatively, then he/she was asked how many standard drinks of any alcoholic beverage he/she had on each day of the past 7 days. The number of days in the past week in which 4 (female) or 5 (male) drinks were consumed was calculated, and a total of 1–2 and >3 days in the past 7 days were considered infrequent and frequent heavy drinking respectively. Those who have ever consumed alcohol but were neither an infrequent or frequent heavy drinker were considered to be non-heavy drinkers. A three-category variable was created: (a) lifetime abstainer or non-heavy drinker; (b) infrequent heavy drinker; and (c) frequent heavy drinker^30^. Asthma and diabetes were based solely on self-reported lifetime diagnosis. For angina, in addition to a self-reported diagnosis, a symptom-based diagnosis based on the Rose questionnaire was also used^31^. BMI was based on self-reported weight and height, and was calculated as weight in kilograms divided by height in meters squared. BMI was categorized as <18.5 (underweight), 18.5–24.9 (normal weight), 25.0–29.9 (overweight), and ≥30 (obese) kg/m^2^ ^32^.

### Statistical analysis

The statistical analysis was performed with Stata 14.1 (Stata Corp LP, College station, Texas). The age- and sex-adjusted prevalence of arthritis for each country was estimated by using the United Nations population pyramids for the year 2010 (http://esa.un.org/wpp/Excel-Data/population.htm) as the standard population using age strata of 18–34, 35–59, and ≥60 years. The subsequent analyses used the overall sample including all countries or samples by country-income level (i.e. low-income or middle-income countries). Multivariable binary logistic regression analysis with arthritis as the outcome was performed to assess the correlates of arthritis. The correlates considered were sex, age, education, wealth, setting, smoking, alcohol consumption, angina, asthma, diabetes, and BMI. In order to assess the association between arthritis (exposure variable) and the mental health outcomes, multivariable binary and multinomial logistic regression analyses were conducted. Multinomial logistic regression analysis was conducted for psychosis and depression type, which consisted of more than two categories. When anxiety, sleep problems, and perceived stress were the outcome, binary logistic regression analysis was conducted. These analyses adjusted for all the potential correlates mentioned above as they have been reported to be associated with both arthritis and mental health outcomes^12, 33–37^. All variables were included in the models as categorical variables with the exception of age (continuous variable). All regression analyses were adjusted for country by including dummy variables for each country in the models, as in previous WHS publications^38, 39^. We did not use multilevel models as multilevel analyses with complex study designs can produce potentially biased estimates^40^. Turkey was not included in the regression analyses as it lacked data on education. Furthermore, due to completely missing data, the analysis with perceived stress as the outcome did not include Brazil, Hungary, and Zimbabwe, while Morocco was omitted from the analysis with anxiety as the outcome. The sample weighting and the complex study design were taken into account in all analyses with the use of the Stata *svy* command. Results from the logistic regression models are presented as odds ratios (ORs) with 95% confidence intervals (CIs). The level of statistical significance was set at P < 0.05.

Less than 5% of the data was missing for all variables used in the analysis with the exception of education (8.1%), wealth (8.4%), alcohol consumption (5.2%), BMI (30.1%), diabetes (15.9%), and psychosis (15.8%). For the regression analyses, we conducted multiple imputation of missing values using the *mi* commands in Stata using chained equations (20 imputations)^41^. This method uses information from all other variables except the one being imputed to impute missing values. The variables included in the imputation model were the outcome and all other covariates^42^. A predictive mean matching algorithm was used for continuous variables, while for dichotomous and ordinal variables, binary logistic regression models and ordered logistic regression models, respectively, were used. The results based on complete case analysis were similar (Appendixs 2 and 3).

## Results

**Figure 1:**
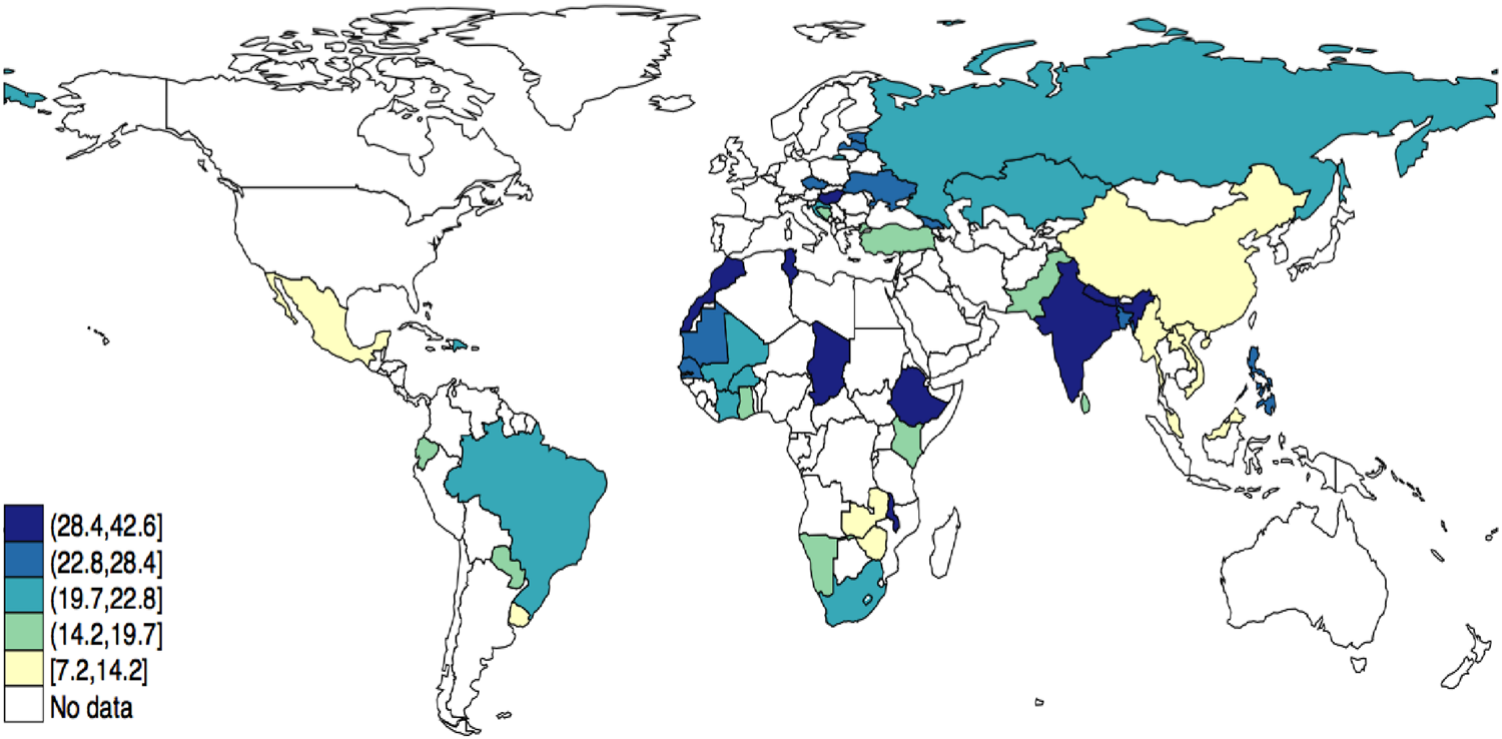
Age- and sex-adjusted prevalence (%) of arthritis. Estimates were calculated using the United Nations population pyramids for the year 2010. The figure was created with STATA 13.1 (StataCorp. 2013. Stata Statistical Software: Release 13. College Station, TX: StataCorp LP).

A total of 245,706 individuals (LIC = 102,211 and MIC = 143,495) constituted the final analytical sample. Overall, there were more females than males (50.7% vs. 49.3%), and the mean (SD) age was 38.4 (16.0) years. The sample size of each country ranged from 929 (Latvia) to 38,746 (Mexico) (Table 1). The age- and sex-adjusted prevalence of arthritis in the overall sample was 22.4% (95%CI = 21.9%–22.8%) with the corresponding figures for low-income and middle-income countries being 23.4% (95%CI = 22.7%–24.1%) and 20.9 (95%CI = 20.4%–21.9%) respectively. This figure ranged from 7.2% (Myanmar) to 42.6% (Malawi) with high prevalence also being observed in Chad (40.6%), Morocco (34.7%), and India (32.4%) (Table 1, Fig. 1). The sample characteristics are shown in Table 2. In the overall sample, female sex, older age, lower education, poverty, rural setting, smoking, lower alcohol consumption, angina, asthma, diabetes, higher BMI, and all the mental health outcomes were more common among those with arthritis (Table 2).

**Table 1:** Sample size and age- and sex-adjusted prevalence of arthritis by country.

Country	Low-income countries			Middle-income countries	
	Unweighted N	% (SE)	Country	Unweighted N	% (SE)
Bangladesh	5,942	24.0 (1.1)	Bosnia Herzegovina	1,031	18.8 (1.8)
Burkina Faso	4,948	20.2 (1.4)	Brazil	5,000	20.6 (0.7)
Chad	4,870	40.6 (1.4)	China	3,994	14.2 (1.1)
Comoros	1,836	18.5 (1.4)	Croatia	993	22.8 (1.4)
Ethiopia	5,089	31.2 (1.2)	Czech Republic	949	24.0 (1.9)
Ghana	4,165	19.0 (0.8)	Dominican Republic	5,027	20.1 (0.8)
India	10,687	32.4 (1.3)	Ecuador	5,675	16.9 (0.9)
Ivory Coast	3,251	21.5 (1.2)	Estonia	1,020	27.4 (1.7)
Kenya	4,640	15.4 (0.9)	Georgia	2,950	23.0 (0.9)
Laos	4,988	12.9 (0.7)	Hungary	1,419	30.1 (1.2)
Malawi	5,551	42.6 (1.1)	Kazakhstan	4,499	21.7 (0.8)
Mali	4,886	19.9 (0.9)	Latvia	929	24.2 (1.7)
Mauritania	3,902	28.4 (1.2)	Malaysia	6,145	14.2 (0.5)
Myanmar	6,045	7.2 (0.6)	Mauritius	3,968	15.2 (0.9)
Nepal	8,820	30.1 (0.6)	Mexico	38,746	9.7 (0.3)
Pakistan	6,501	19.7 (0.8)	Morocco	5,000	34.7 (1.1)
Senegal	3,461	27.3 (1.2)	Namibia	4,379	15.5 (1.0)
Vietnam	4,174	9.4 (0.9)	Paraguay	5,288	18.5 (0.6)
Zambia	4,165	9.0 (0.7)	Philippines	10,083	27.3 (0.8)
Zimbabwe	4,290	11.8 (0.7)	Russia	4,427	22.5 (1.2)
			South Africa	2,629	20.9 (1.3)
			Sri Lanka	6,805	14.7 (0.8)
			Tunisia	5,202	30.9 (0.9)
			Turkey	11,481	19.1 (0.7)
			Ukraine	2,860	25.1 (1.3)
			Uruguay	2,996	14.1 (0.5)

Abbreviation: SE standard error.

All age- and sex-adjusted weighted estimates were calculated using the United Nations population pyramids for the year 2010.

**Table 2:** Sample characteristics (overall and by country-income level and the presence of arthritis).

Characteristic	Category	Overall			Low-income countries			Middle-income countries		
		Total	Arthritis		Total	Arthritis		Total	Arthritis	
			No	Yes		No	Yes		No	Yes
		49.3	51.9	39.0	50.5	52.9	41.4	47.7	50.6	35.8
	Female	50.7	48.1	61.0	49.5	47.1	58.6	52.3	49.4	64.2
Age (years)	Mean	38.4	35.9	48.1	36.8	34.6	45.1	40.5	37.6	51.8
	(SD)	(16.0)	(14.8)	(16.8)	(15.3)	(14.1)	(16.8)	(16.6)	(15.4)	(16.4)
Education^a^	No formal	26.5	23.4	38.4	40.6	36.4	56.6	7.0	5.5	13.2
	≤Primary	30.9	30.9	30.8	32.9	34.0	28.7	28.1	26.8	33.7
	Secondary completed	33.4	36.0	23.4	20.5	22.8	11.5	51.2	54.2	39.7
	Tertiary completed	9.2	9.6	7.5	6.0	6.8	3.1	13.6	13.5	13.5
Wealth	Poorest	20.1	19.3	23.5	20.2	19.6	23.0	20.1	19.0	24.2
	Poorer	20.1	19.4	22.7	20.0	19.2	22.8	20.1	19.6	22.5
	Middle	20.0	20.0	20.0	19.9	19.8	20.0	20.2	20.3	20.1
	Richer	19.8	20.4	17.6	20.0	20.5	18.0	19.7	20.3	17.3
	Richest	20.0	20.9	16.2	20.0	20.9	16.2	19.9	20.9	16.1
Setting	Rural	55.7	54.6	59.5	74.8	73.8	80.6	31.0	30.5	32.7
	Urban	44.3	45.4	40.5	25.2	26.2	19.4	69.0	69.5	67.3
Smoking	No	73.1	73.4	72.0	73.3	74.4	68.6	72.9	72.1	76.3
	Yes	26.9	26.6	28.0	26.7	25.6	31.4	27.1	27.9	23.7
Alcohol	Never/non-heavy	95.4	95.1	96.3	97.8	97.9	97.5	92.4	91.9	94.7
consumption	Infrequent heavy	3.6	3.8	2.8	1.5	1.5	1.6	6.2	6.7	4.3
	Frequent heavy	1.0	1.0	0.9	0.7	0.7	0.8	1.4	1.5	1.0
Angina	No	85.7	90.5	66.8	85.2	89.7	67.5	86.3	91.4	65.9
	Yes	14.3	9.5	33.2	14.8	10.3	32.5	13.7	8.6	34.1
Asthma	No	94.9	95.9	91.1	95.9	97.0	91.8	93.7	94.6	90.2
	Yes	5.1	4.1	8.9	4.1	3.0	8.2	6.3	5.4	9.8
Diabetes	No	97.0	97.9	93.7	98.1	98.6	95.8	95.5	96.8	90.8
	Yes	3.0	2.1	6.3	1.9	1.4	4.2	4.5	3.2	9.2
BMI (kg/m^2^)	18.5–24.9	57.6	59.3	50.5	62.1	63.1	57.7	53.3	55.6	43.9
	25.0–29.9	19.8	19.2	22.6	11.5	11.2	13.0	27.9	27.0	31.6
	≥30	9.1	8.0	13.7	6.1	5.7	7.7	12.0	10.3	19.2
	<18.5	13.5	13.5	13.2	20.3	20.0	21.6	6.8	7.1	5.4
Psychosis	Symptom (−) Diagnosis (−)	85.0	87.3	75.8	85.9	88.4	75.7	83.7	85.7	76.0
	Symptom (+) Diagnosis (−)	13.9	11.9	21.8	12.9	10.7	21.6	15.4	13.6	22.1
	Diagnosis (+)	1.1	0.8	2.3	1.2	0.9	2.7	0.9	0.6	1.9
Depression type	No depression	88.4	91.3	75.6	88.3	91.3	74.5	88.5	91.2	77.1
	Subsyndromal depression	2.4	1.9	4.8	3.0	2.3	6.2	1.6	1.3	2.8
	Brief depressive episode	2.7	2.2	5.0	2.3	1.9	4.2	3.4	2.7	6.1
	Depressive episode	6.5	4.6	14.6	6.4	4.5	15.1	6.6	4.8	13.9
Anxiety^b^	No	88.4	90.6	79.8	90.9	93.2	82.2	85.2	87.4	76.6
	Yes	11.6	9.4	20.2	9.1	6.8	17.8	14.8	12.6	23.4
Sleep problems	No	92.3	94.9	81.8	93.0	95.4	83.7	91.3	94.3	79.3
	Yes	7.7	5.1	18.2	7.0	4.6	16.3	8.7	5.7	20.7
Perceived stress^c^	No	80.5	83.0	70.9	76.0	78.5	66.4	88.9	91.4	79.3
	Yes	19.5	17.0	29.1	24.0	21.5	33.6	11.1	8.6	20.7

Abbreviation: SD standard deviation; BMI body mass index.

Data are % unless otherwise stated.

All estimates are based on weighted sample.

^a^Turkey is not included as it lacked information on education.

^b^Morocco is not included as it lacked information on anxiety.

^c^Brazil, Hungary, and Zimbabwe are not included as they lacked information on perceived stress.

The correlates of arthritis estimated by multivariable binary logistic regression are presented in Table 3. In the overall sample, female sex, older age, lower education, poverty, smoking, angina, asthma, diabetes, and higher BMI were associated with arthritis. Similar correlates were found in the samples stratified by country-income level although there were some differences. Specifically, wealth and BMI were not significantly associated with arthritis in low-income countries.

**Table 3:** Correlates of arthritis assessed by multivariable binary logistic regression analysis.

Characteristic	Overall		Low-income countries		Middle-income countries	
	OR	95%CI	OR	95%CI	OR	95%CI
**Sex**						
Male	1.00		1.00		1.00	
Female	1.58***	[1.50,1.67]	1.56***	[1.45,1.69]	1.62***	[1.51,1.74]
**Age** (years)	1.04***	[1.04,1.04]	1.04***	[1.04,1.04]	1.04***	[1.04,1.05]
**Education**						
No formal	1.00		1.00		1.00	
≤Primary	0.95	[0.88,1.01]	0.94	[0.86,1.02]	0.95	[0.84,1.07]
Secondary completed	0.75***	[0.69,0.82]	0.71***	[0.63,0.80]	0.82**	[0.72,0.94]
Tertiary completed	0.69***	[0.62,0.78]	0.62***	[0.51,0.76]	0.78**	[0.66,0.92]
**Wealth**						
Poorest	1.00		1.00		1.00	
Poorer	1.04	[0.97,1.13]	1.08	[0.97,1.20]	0.98	[0.88,1.08]
Middle	0.97	[0.90,1.05]	0.98	[0.88,1.09]	0.94	[0.85,1.04]
Richer	0.90*	[0.82,0.98]	0.92	[0.81,1.04]	0.86**	[0.76,0.96]
Richest	0.86**	[0.78,0.95]	0.88	[0.76,1.01]	0.84**	[0.74,0.95]
**Setting**						
Rural	1.00		1.00		1.00	
Urban	0.94	[0.87,1.01]	0.93	[0.82,1.05]	0.95	[0.87,1.04]
**Smoking**						
No	1.00		1.00		1.00	
Yes	1.16***	[1.08,1.23]	1.17**	[1.07,1.29]	1.16***	[1.07,1.26]
**Alcohol consumption**						
Never/non-heavy	1.00		1.00		1.00	
Infrequent heavy	1.01	[0.89,1.16]	1.26	[0.97,1.64]	0.94	[0.81,1.09]
Frequent heavy	0.96	[0.77,1.20]	1.17	[0.88,1.55]	0.82	[0.59,1.15]
**Angina**	2.90***	[2.71,3.10]	2.86***	[2.59,3.15]	2.92***	[2.69,3.17]
**Asthma**	1.49***	[1.34,1.66]	1.49***	[1.28,1.75]	1.50***	[1.31,1.73]
**Diabetes**	1.58***	[1.38,1.82]	1.85***	[1.45,2.36]	1.39***	[1.18,1.64]
**BMI** (kg/m^2^)						
18.5–24.9	1.00		1.00		1.00	
25.0–29.9	1.12**	[1.05,1.21]	1.06	[0.95,1.19]	1.21***	[1.11,1.32]
≥30.0	1.34***	[1.20,1.50]	1.17	[0.97,1.42]	1.56***	[1.39,1.74]
<18.5	0.99	[0.91,1.08]	0.97	[0.87,1.08]	1.02	[0.89,1.16]

Abbreviation: OR odds ratio; CI confidence interval; BMI Body mass index.

The models are adjusted for all variables in the table and country.

Turkey is not included in the regression analyses as it lacked information on education.

*p < 0.05, **p < 0.01, ***p < 0.001.

### Mental health comorbidity and arthritis

The associations between arthritis and mental health outcomes estimated by multinomial and logistic regression are illustrated in Table 4. In the overall sample, after adjusting for potential confounders, having arthritis increased the odds of having subclinical psychosis (OR = 1.85; 95%CI = 1.72–1.99) or a psychosis diagnosis (OR = 2.48; 95%CI = 2.05–3.01) compared to no psychosis. The odds for all types of depression compared to no depression were also increased in those with arthritis: subsyndromal depression (OR = 1.92; 95%CI = 1.64–2.26); brief depressive episode (OR = 2.14; 95%CI = 1.88–2.43); depressive episode (OR = 2.43; 95%CI = 2.21–2.67). Furthermore, arthritis was also associated with increased odds for anxiety (OR = 1.75; 95%CI = 1.63–1.88); sleep problems (OR = 2.23; 95%CI = 2.05–2.43) and perceived stress (OR = 1.43; 95%CI = 1.33–1.53). The results for middle-income and low-income countries were similar.

**Table 4:** The association between arthritis and mental health outcomes estimated by multinomial and binary logistic regression.

Outcome	Overall		Low-income countries		Middle-income countries	
	OR	95%CI	OR	95%CI	OR	95%CI
*Multinomial logistic regression*						
Psychosis						
Symptom (−) Diagnosis (−)	1.00		1.00		1.00	
Symptom (+) Diagnosis (−)	1.85***	[1.72,1.99]	1.77***	[1.59,1.97]	1.98***	[1.79,2.18]
Diagnosis (+)	2.48***	[2.05,3.01]	2.27***	[1.77,2.91]	2.98***	[2.24,3.97]
Depression type						
No depression	1.00		1.00		1.00	
Subsyndromal depression	1.92***	[1.64,2.26]	1.89***	[1.55,2.30]	2.06***	[1.59,2.67]
Brief depressive episode	2.14***	[1.88,2.43]	2.09***	[1.75,2.49]	2.23***	[1.86,2.68]
Depressive episode	2.43***	[2.21,2.67]	2.39***	[2.10,2.73]	2.51***	[2.21,2.83]
*Binary logistic regression*						
Anxiety^a^	1.75***	[1.63,1.88]	1.69***	[1.53,1.86]	1.84***	[1.65,2.05]
Sleep problems	2.23***	[2.05,2.43]	2.25***	[1.99,2.55]	2.21***	[1.98,2.47]
Perceived stress^b^	1.43***	[1.33,1.53]	1.40***	[1.28,1.52]	1.49***	[1.35,1.65]

Abbreviation: OR odds ratio; CI confidence interval.

All models are adjusted for sex, age, education, wealth, setting, smoking, alcohol consumption, angina, asthma, diabetes, BMI, and country.

Turkey is not included in the regression analyses as it lacked information on education.

^a^Morocco is not included as it lacked information on anxiety.

^b^Brazil, Hungary, and Zimbabwe are not included as they lacked information on perceived stress.

***p < 0.001.

## Discussion

The current large scale study involving almost a quarter of a million people over 46 LMICs established that the age- and sex-adjusted prevalence of arthritis was 22.4% across all countries and 23.4% in low-income and 20.9% in middle-income countries. In the overall sample, the correlates of arthritis estimated by multivariable logistic regression included female sex, older age, lower education, poverty, smoking, angina, asthma, diabetes, and higher BMI. In LMICs collectively, we observed that people with arthritis were consistently more likely to have depression (all subtypes), subclinical psychosis, established psychotic disorder, sleep problems, anxiety, and higher levels of perceived stress. The increased mental health comorbidity among those with arthritis was consistently raised in those in both low-income and middle-income countries.

The prevalence of arthritis (22.3%) found in our LMIC sample was higher than a previous meta-analysis on the prevalence of RA which reported that 0.16% (95%CI: 0.11–0.20%) and 0.75% (95%CI: 0.60–0.90%) of males and females respectively had RA^14^. Moreover, this is also higher than the prevalence of OA reported in a recent global burden of disease survey of 3% and 5% in males and females respectively in Africa and central Asia^29^. However, a previous study using similar definitions of arthritis as in our study have found similarly high figures in 9 LMICs, although the sample was limited to adults aged 50 years or older^18^. The potential reason for this might be the self-report questions used in the WHS in addition to a reflection in a potentially heightened prevalence of pain and stiffness among the sample.

In accordance with the literature in high-income countries^6, 8–10^, we observed heightened odds for all depression subtypes among those with arthritis versus controls. In many ways, the heightened mental health comorbidity among those with arthritis is unsurprising given the potential impact of pain, disability, and pressure to continue earning to provide an income for the family. It is interesting to note that all depression subtypes were associated with heightened odds with arthritis and to the best of our knowledge, our paper is the first multinational paper to consider this relationship. This in contrast to recent work among people with back pain in LMICs, where an incremental increased odds of depression was noted with more severe depression subtypes^43^. It is also perhaps of little surprise that people with arthritis were more likely to have anxiety, sleep disturbances, and perceived stress. These relationships may potentially be explained by increased levels of pain associated with arthritis. Specifically, the underlying shared pathophysiology of pain and depression could account for this, since both depression and pain facilitate modulation in the periaqueductal gray, amygdala, and hypothalamus regions^44, 45^. Second, arthritis^46^, pain, and depression^47^ are associated with and exacerbated by low levels of physical activity and social isolation^48, 49^. Thus, it is possible that these factors, which were not assessed in the current study, are implicated in the link between arthritis and worse mental health. Increasing physical activity has established efficacy in reducing both depression^50, 51^ and pain as well as their associated disability^52^ and could therefore be key to reducing the burden of this comorbidity and improving function. Finally, within the context of some LMICs, the high prevalence of HIV and tuberculosis^53^ may account for both the depression^54^ and pain associated with arthritis^55^.

The increased odds of arthritis among people with a diagnosis of psychosis has to the best of our knowledge not been previously reported in LMICs, and data on the relation between subclinical psychosis and arthritis is scarce. Furthermore, very few community-based multinational studies exist on these associations even in high-income countries. Our finding that arthritis is associated with higher odds for psychotic disorders (e.g. schizophrenia) is in contrast to the trend that has previously been reported. A recent meta-analysis with polygenic risk score analysis found that people with schizophrenia are at reduced odds of RA, although this does not appear to be related to polygenic risk scores and may be more related to environmental risk factors such as the anti-inflammatory effects of antipsychotic medication^56^. A nationally representative study in Taiwan found that people with schizophrenia were not more likely than controls to develop OA (HR = 0.89; 95%CI = 0.81–1.01), p = 0.53)^57^. Thus, it appears that people with psychosis in LMICs may be more likely to have arthritis compared to their high-income counterparts. The precise reasons for this are unclear and future studies are warranted to assess whether our results may be replicated. RA aside, there is evidence that any history of autoimmune disease (including psoriasis, which can have a significant joint component) can increase the odds of developing schizophrenia^58^. In terms of the heightened odds for subclinical psychosis in arthritis, this may be explained by the psychological distress caused by the symptoms of arthritis (e.g. pain) which has been associated with psychotic symptoms in the general population^30, 59, 60^.

It is established from studies conducted in high-income countries that comorbid mental health outcomes among those with arthritis are associated with worse pain and poorer treatment outcomes^8, 10, 61^. Thus clearly integrated mental and physical healthcare is essential. However, in the context of arthritis, there is a paucity of evidence-based literature, in particular intervention studies. Regardless, the current data have important public health implications, particularly since the data were multi-national, population-based, and predominantly nationally representative, rather than most literature to date which is derived from clinical samples. Understanding and treating comorbid mental health outcomes among those with arthritis is essential. An important environmental barrier in the care of people with mental and physical health problems in LMICs is the lack of integrated mental and physical healthcare services and the poorly developed community-based psychiatric services^62^. Closer integration of primary and mental health care in these countries is needed, but without obscuring the responsibility for arthritis and mental health assessment, prevention and management^63^. We suggest that people with arthritis are assessed for the mental health conditions assessed in our study (i.e., depression, anxiety, sleep disturbance, stress and psychosis) so that appropriate interventions can be provided. More research is required to understand the mental health burden of arthritis in LMICs and context-specific trials and evaluations in LMICs therefore are urgently needed.

Some study design limitations need to be considered. First, the categorization of arthritis was based on self-report and it was not possible to explore in more detail the type, nature, and severity of arthritis. In particular, it was not possible to differentiate between OA and RA and other forms of arthritis. Thus, clearly, future research is required to disentangle the nature and mental health impact of the different forms of arthritis in LMICs. Second, the study sample only included non-institutionalized individuals. Thus, those with severe mental disorders or arthritis could have been omitted from the sample, leading to an underestimate of the associations. Next, we adjusted for a variety of potential confounders but we cannot preclude the possibility of residual confounding. For example, we were only able to adjust for a limited number of physical comorbidities. Finally, the data is cross-sectional thus it is not possible to disentangle the directionality of the relationships observed. It is important that future research attempts to understand the underlying explanatory factors of the relationships we observed. In particular, research considering psychotic disorders and RA are required among LMICs.

In conclusion, our large community-based study has demonstrated that arthritis is associated with a broad range of elevated mental health comorbidity. For the first time we demonstrated on a multinational scale that depression subtypes, psychosis spectrum, stress sensitivity and anxiety are increased in those with arthritis. Future longitudinal research is required to elucidate the course, trajectory, and outcomes of comorbid mental health and arthritis in LMICs.

## Electronic supplementary material

Appendix
