# Supplementary material for: Lifetime self-reported arthritis is associated with elevated levels of mental health burden: A multi-national cross sectional study across 46 low- and middle-income countries
Source: Sci Rep. 2017 Aug 2;7:7138. doi: 10.1038/s41598-017-07688-6 (PMC5541038; doi:10.1038/s41598-017-07688-6)
Supplement: Supplementary file 1 — Appendix [file 41598_2017_7688_MOESM1_ESM.pdf]

Appendix 1 of ethical approval committees for the World health survey

Ethical approval

Ethical approval was obtained from each of the following committees in the respective countries for the World Health Survey:

Country	Ethical committee approving the study
Bangladesh	Mitra and Associates
Bosnia Herzegovina	The Federal Public Health Institute
Brazil	Fundacao Oswaldo Cruz
Burkina Faso	Institut de Recherche en Sciences de la Santé
Chad	Faculté des Sciences de la Santé, Univ N'Djamena
China	Centre for Health Statistics Information
Comoros	Bureau Comorien de Conseil
Congo	Unité de recherche sur les systèmes de santé
Ivory Coast	Ministère de la Santé
Croatia	The Croatian National Institute of Public Health
Czech Republic	Institute of Health Information and Statistics
Dominican Republic	Centro de Estudios Sociales y Demográficos (CESDEM)
Ecuador	Fundación Ecuatoriana para la Salud y el Desarroll (FESALUD)
Estonia	Saar Poll Ltd.
Ethiopia	Department of Community Health, Jimma University
Georgia	Georgian State Medical Academy (GSMA)
Ghana	Department of Community Health, Ghana Medical Sch
Hungary	Johan Bela National Centre for Epidemiology
India	International Institute of Population Sciences
Kazakhstan	Kazakstan School of Public Health (KSPH)
Kenya	Central Bureau of Statistics
Laos	National Institute of Public Health, Ministry of Health
Latvia	The Health Promotion Center
Malawi	Centre for Social Research (CSR)
Malaysia	Public Health Institute, Ministry of Health
Mali	Cellule de Planification et de Statistique, (CPS)
Mauritania	Office Nationale de la Statistique (ONS)
Mauritius	Mauritius Instituteof Health
Mexico	Instituto Nacional de Salud Pública

Morocco	Ministère de la Santé
Myanmar	Department of Medical Research, Ministry of Health
Namibia	Ministry of Health
Nepal	ORG-MARG Nepal PVT.Ltd
Pakistan	Ministry of Health
Paraguay	Fac.de Ciencias Veterinarias, Univ. Nacional/DGEEC
Philippines	College of Medicine, University of the Philippines
Russia	Semashko Institute for Research on Social Hygiene,
Senegal	Direction Etudes, Recherche et Formation (DERF)
Slovakia	Environment,s.c., Centre of Biostatistics and Envi
South Africa	Community Agency for Social Enquiry (CASE)
Sri Lanka	Ministry of Health
Swaziland	Faculty of Health Sciences, Univ Swaziland
Tunisia	Institut National de la Santé Publique
Ukraine	Odessa State Medical University
Uruguay	Centro de Estudios de Economia y Salud (CEES)
Vietnam	Ministry of Health
Zambia	School of Humanities & Social Sciences, University of Zambia
Zimbabwe	Community Health, University of Zimbabwe

Web Appendix 2 Correlates of arthritis assessed by multivariable binary logistic regression analysis (Complete case analysis)

Characteristic	Overall (N=147,732) OR	95%CI	Low-income countries (N=56,896) OR	95%CI	Middle-income countries (N=90,836) OR	95%CI
**Sex**						
Male	1.00		1.00		1.00	
Female	1.65***	[1.53,1.77]	1.68***	[1.48,1.90]	1.64***	[1.51,1.79]
**Age** (years)	1.04***	[1.04,1.04]	1.04***	[1.04,1.04]	1.04***	[1.04,1.05]
**Education**						
No formal	1.00		1.00		1.00	
≤Primary	1.01	[0.91,1.12]	1.06	[0.93,1.21]	0.89	[0.77,1.04]
Secondary completed	0.82**	[0.73,0.93]	0.83*	[0.69,1.00]	0.75**	[0.63,0.89]
Tertiary completed	0.75***	[0.65,0.87]	0.72**	[0.56,0.92]	0.71***	[0.58,0.86]
**Wealth**						
Poorest	1.00		1.00		1.00	
Poorer	1.07	[0.98,1.18]	1.20*	[1.04,1.39]	0.96	[0.85,1.08]
Middle	1.02	[0.93,1.13]	1.09	[0.93,1.27]	0.96	[0.85,1.07]
Richer	0.91	[0.83,1.01]	0.96	[0.83,1.12]	0.85*	[0.75,0.97]
Richest	0.87*	[0.78,0.98]	0.94	[0.78,1.12]	0.81**	[0.70,0.95]
**Setting**						
Rural	1.00		1.00		1.00	
Urban	0.89**	[0.83,0.97]	0.86*	[0.75,0.99]	0.94	[0.85,1.03]
**Smoking**						
No	1.00		1.00		1.00	

Yes	1.24***	[1.14,1.34]	1.26**	[1.09,1.44]	1.24***	[1.13,1.35]
**Alcohol consumption**						
Never/non-heavy	1.00		1.00		1.00	
Infrequent heavy	0.93	[0.80,1.07]	1.10	[0.79,1.55]	0.89	[0.76,1.05]
Frequent heavy	0.86	[0.65,1.13]	1.18	[0.81,1.71]	0.72	[0.49,1.06]
**Angina**	2.72***	[2.51,2.96]	2.64***	[2.31,3.01]	2.78***	[2.52,3.07]
**Asthma**	1.52***	[1.34,1.74]	1.56***	[1.24,1.96]	1.51***	[1.29,1.76]
**Diabetes**	1.57***	[1.32,1.88]	1.99***	[1.38,2.87]	1.41***	[1.16,1.71]
**BMI** (kg/m^2)						
18.5-24.9	1.00		1.00		1.00	
25.0-29.9	1.19***	[1.09,1.30]	1.10	[0.92,1.31]	1.26***	[1.15,1.39]
≥30.0	1.49***	[1.34,1.66]	1.12	[0.89,1.41]	1.71***	[1.51,1.93]
<18.5	0.93	[0.85,1.03]	0.91	[0.81,1.03]	0.95	[0.81,1.11]

Abbreviation: OR odds ratio; CI confidence interval; BMI Body mass index

The models are adjusted for all variables in the table and country.

Turkey is not included in the regression analyses as it lacked information on education.

* p<0.05, ** p<0.01, *** p<0.001

Web Appendix 3 The association between arthritis and mental health outcomes estimated by multinomial and binary logistic regression (Complete case analysis)

	Overall		Low-income countries		Middle-income countries	
Outcome	OR	95%CI	OR	95%CI	OR	95%CI
Multinomial logistic regression						
Psychosis	N=146,728		N=56,248		N=90,480	
Symptom (-) Diagnosis (-)	1.00		1.00		1.00	
Symptom (+) Diagnosis (-)	1.93***	[1.76,2.10]	1.83***	[1.59,2.11]	2.01***	[1.79,2.26]
Diagnosis (+)	2.64***	[2.04,3.41]	2.56***	[1.76,3.72]	2.80***	[2.01,3.89]
Depression type	N=146,294		N=56,575		N=89,719	
No depression	1.00		1.00		1.00	
Subsyndromal depression	2.11***	[1.75,2.55]	2.13***	[1.63,2.79]	2.08***	[1.62,2.68]
Brief depressive episode	2.29***	[1.93,2.70]	2.23***	[1.74,2.86]	2.35***	[1.89,2.93]
Depressive episode	2.56***	[2.28,2.88]	2.54***	[2.11,3.06]	2.63***	[2.29,3.02]
Binary logistic regression						
Anxiety[a]	N=145,458		N=56,691		N=88,767	
	1.90***	[1.72,2.10]	1.94***	[1.65,2.28]	1.86***	[1.64,2.11]
Sleep problems	N=147,506		N=56,738		N=90,768	
	2.40***	[2.15,2.68]	2.57***	[2.10,3.14]	2.29***	[2.02,2.61]
Perceived stress[b]	N=138,512		N=53,848		N=84,664	
	1.46***	[1.34,1.60]	1.45***	[1.29,1.63]	1.46***	[1.30,1.63]

Abbreviation: OR odds ratio; CI confidence interval

All models are adjusted for sex, age, education, wealth, setting, smoking, alcohol consumption, angina, asthma, diabetes, obesity, and country.

Turkey is not included in the regression analyses as it lacked information on education.

[a] Morocco is not included as it lacked information on anxiety.

[b] Brazil, Hungary, and Zimbabwe are not included as they lacked information on perceived stress.

*** p<0.001
